# Identification of Distinctive Primary Metabolites Influencing Broccoli (*Brassica oleracea*, var. *Italica*) Taste

**DOI:** 10.3390/foods12020339

**Published:** 2023-01-11

**Authors:** Sergio Chevilly, Laura Dolz-Edo, José Blanca, Lynne Yenush, José M. Mulet

**Affiliations:** 1 Instituto de Biología Molecular y Celular de Plantas, Universitat Politècnica de València-Consejo Superior de Investigaciones Científicas, 46022 Valencia, Spain; 2 Instituto Universitario de Conservación y Mejora de la Agrodiversidad Valenciana, COMAV, Universitat Politècnica de València, 46022 Valencia, Spain

**Keywords:** Broccoli, *Brassica oleracea*, taste, consumers, metabolites, GABA

## Abstract

Broccoli (*Brassica oleracea* L. var. *Italica Plenck*) is a cruciferous crop that is considered to be a good source of micronutrients. Better taste is a main objective for breeding, as consumers are demanding novel cultivars suited for a healthy diet, but ones that are more palatable. This study aimed to identify primary metabolites related to cultivars with better taste according to a consumer panel. For this purpose, we performed a complete primary metabolomic profile of 20 different broccoli cultivars grown in the field and contrasted the obtained data with the results of a consumer panel which evaluated the taste of the same raw buds. A statistical analysis was conducted to find primary metabolites correlating with better score in the taste panels. According to our results, sugar content is not a distinctive factor for taste in broccoli. The accumulation of the amino acids leucine, lysine and alanine, together with Myo-inositol, negatively affected taste, while a high content of γ-aminobutyric acid (GABA) is a distinctive trait for cultivars scoring high in the consumer panels. A Principal Component Analysis (PCA) allowed us to define three different groups according to the metabolomic profile of the 20 broccoli cultivars studied. Our results suggest molecular traits that could be useful as distinctive markers to predict better taste in broccoli or to design novel biotechnological or classical breeding strategies for improving broccoli taste.

## 1. Introduction

Broccoli (*Brassica oleracea* var. *Italica*) is a plant that belongs to the *Brassicaceae* family. During the last decades, its importance has increased. In 1980, the global production of broccoli and cauliflower was 5.94 million metric tons, while in 2019 the production increased to 26.9 million metric tons [1], with China and India as the main producers. This increase in production has come as a consequence of increasing consumer demand. One of the reasons explaining this dramatic increase is that broccoli is considered to be part of a healthy diet, as it provides valuable molecules and micronutrients. Broccoli is rich in vitamins C and E, quercetin and kaempferol glycosides [2]. Broccoli is also rich in glucosinolates, a group of about 120 molecules derived from amino acids that have a β-D-glucopyranose residue linked through a sulphur atom to a (Z)-N-hydroximinosulfate ester, plus a variable R group [3]. These molecules confer the characteristic pungent flavor of broccoli. Moreover, some products derived from glucosinolate hydrolisis, such as sulforaphane, may reduce the risk of lung, breast, gastric, prostate, and kidney cancer [4,5].

Broccoli is not very popular among some consumers, especially children [6]. Some adult consumers also dislike broccoli, and this could be explained by genetic variations related to the capsaicin receptor TRPV1, which may render some populations very sensitive to components present in broccoli, thus explaining the aversion [7]. Studies on organoleptic and chemical properties of broccoli buds have been carried out in order to meet consumer demands and to promote the consumption of healthy foods. Different broccoli cultivars diverge in external and internal sensory attributes. Among the factors that influence consumer preferences there are visual aspects (color), taste (bitter and sweet), and flavor aspects. Flavor has been defined as a mingled but unitary experience which includes sensations of taste, smell, and pressure, and often cutaneous sensations such as warmth, color, or mild pain. Flavor depends on different parameters, including aromatic volatiles, and especially the sugar/acid ratio [8,9]. In broccoli, flavor has been described as green/grassy, spicy, broccoli-like, cabbage-like, cauliflower-like, kohlrabi-like, and leek-like and mouth-feel pungent [10].

Important differences have been described in terms of taste and flavor among broccoli cultivars. The molecular mechanism underlying these large differences may be explained, at least in part, by changes in the metabolic profile. To confirm this hypothesis, we analyzed the buds of 20 different broccoli cultivars grown in field. In parallel, the organoleptic characteristics of the buds were evaluated by a consumer panel. Our analysis has identified a small group of molecules which correlate with the qualification of different cultivars in the sensory panel that may constitute good targets for future strategies of breeding broccoli for better taste.

## 2. Materials and Methods

### 2.1. Plant Material and Treatments

**Figure 1:**
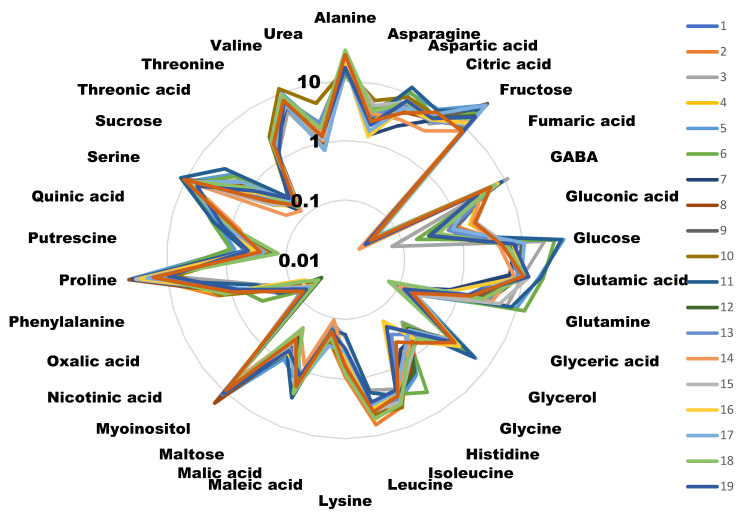
Radial diagram of the metabolite amounts of the different cultivars. Values are the area of the peak per mg of sample represented in a decimal logarithmic scale. Measures are the average of four independent biological replicates. Different colors represent different cultivars. For clarity, error bars have not been represented, but in most of the cases they represent less than 5% of the total value.

This study was performed using twenty different broccoli cultivars (single-crossed hybrids) provided by SAKATA. All of the cultivars used in this study are precommercial, not available in the market and codified by a single number (Figure 1). Plants were grown in field conditions following common procedures reported in the literature for this species [11]. Specifically, to avoid variability due to different environmental or cultivation conditions, we used 40 plants per plot and two repetitions of each plot. All cultivars were cultivated in the same conditions and location. The sowing date was November 2020 and the planting date was December 2020. As plots approached maturity, crops were evaluated every 2–5 days so that samples were collected for the tasting study when they reached their optimal commercial state. The harvesting and evaluation dates were: 9 March, 16 March, 23 March, 29 March, and 6 April, depending on the cultivar. Three heads of each cultivar were sampled at random. Florets were cut from the stem and tasted raw. Aroma and flavor characteristics were given a subjective score from 1 to 5, with 5 = highest quality and 1 = lowest quality. The samples were then frozen in liquid nitrogen for the metabolomic analysis in order to ensure that the metabolic content of the analyzed buds corresponded to those whose taste had been evaluated.

### 2.2. Metabolite Analysis

Frozen buds were lyophilized and then ground with a mechanical tissue disruptor in the presence of liquid nitrogen. 10 mg of sample powder was used for each replicate. The method used in this report was previously described in [12]. We used four biological replicates. Buds were dried by evaporation and the subsequent extraction was performed using methanol and cloromethane.

Derivatization and injection was performed as described in [12]. We collected the mass spectra at 6.25 spectra s^−1^ in the *m*/*z* range 70–800. The ionization energy of 70 eV was used. For the evaluation of the mass spectra we used the CHROMATOF program (LECO, St. Joseph, MI, USA).

### 2.3. Statistical Analysis

Correlation analysis and graphics for this project were generated using Excel software (Microsoft, 2022). A PCA was carried out using the Singular Value Decomposition algorithm implemented by the sckit learn library after standardizing the data using the sckit learn standard scaler [13].

## 3. Results

### 3.1. Determination of the Metabolite Content

Samples from the 20 cultivars included in the study were analyzed for their metabolic content (Figure 1). The histograms for each metabolite in each cultivar, with its error bars, can be found in Supplementary File S1. 

**Figure 2:**
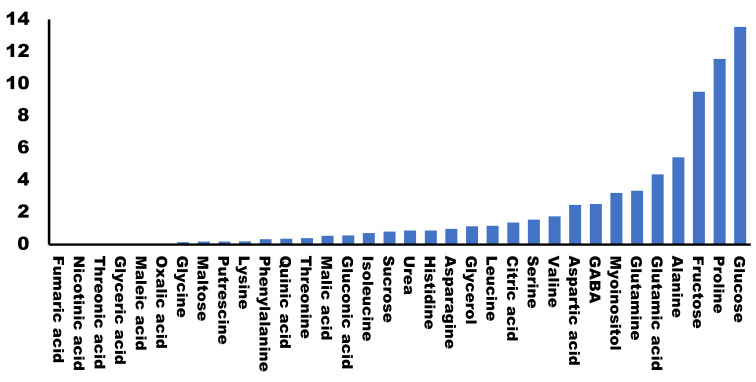
Variability of primary metabolites in different broccoli cultivars. Graphic representation of the standard deviation calculated for the values (area of the peak per mg of sample) of each metabolite in different cultivars.

All plants were cultured and harvested under similar conditions. Some metabolite concentrations were very stable among the different cultivars, while others presented large variations. We evaluated this natural variability by calculating the standard deviation for the different concentrations obtained for each metabolite in each cultivar (Figure 2). A total of 79.5% of the metabolites analyzed presented standard deviations lower than 2, indicating that most of the primary metabolites analyzed were stable among the varieties. On the other hand, we found the highest variability in the concentrations of glucose, proline and fructose (>9).

### 3.2. Correlations between Metabolites and Tasting Score

**Figure 3:**
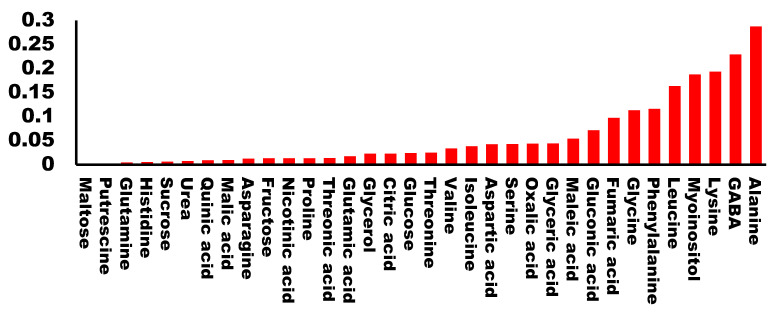
Correlation coefficient (R^2^) between each analyzed metabolite and the tasting score.

Raw buds from the 20 cultivars were evaluated by a panel for their taste properties and assigned a qualification which ranged from 1 to 6 (1 being the lowest and 6 the highest). We performed a regression analysis of the qualification for each cultivar with the amount of each metabolite. As expected, we found very low values of R^2^ for most of the molecules analyzed. Specifically, 81% of the metabolites presented an R^2^ < 0.1 suggesting that no correlation exists between the metabolite levels and the taste of the buds. Only five metabolites presented an R^2^ higher than 0.15. Among them, there were two hydrophobic amino acids (alanine and leucine), one charged amino acid (lysine), one non-proteinogenic amino acid (GABA), and the cyclic polyalcohol myo-inositol (Figure 3). All of the calculated regressions can be found in Supplementary File S2. 

**Figure 4:**
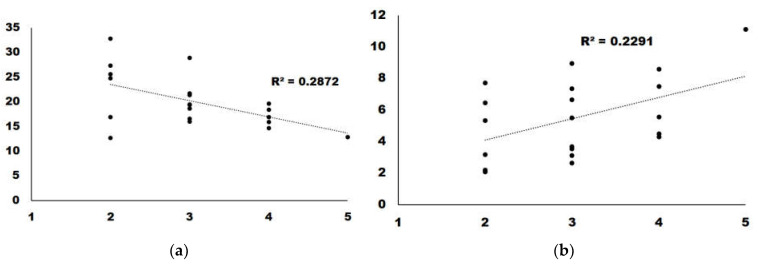
Regression analysis for the two metabolites with the highest correlation: (**a**) Alanine; (**b**) GABA.

We analyzed the correlations with a score higher than 0.15. GABA presented a regression slope higher than 0, which indicates a positive correlation between GABA content and taste. For the remaining four, the regression slope was <0, indicating that a higher amount of metabolite correlated with a lower score in the taste panel (Figure 4).

### 3.3. Principal Component Analysis (PCA) of the Data

**Figure 5:**
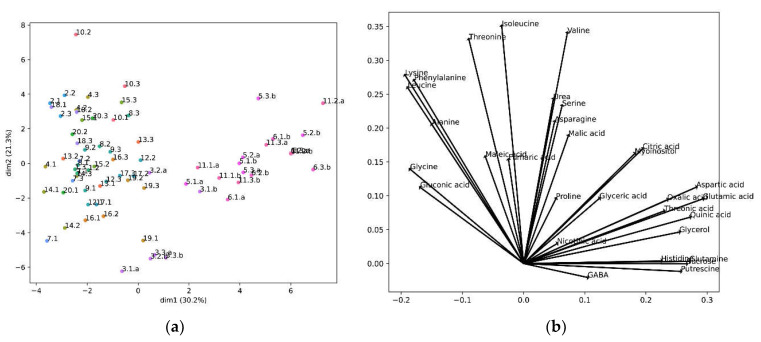
PCA results: (**a**) PCA projections of the cultivars in the first two principal components. The percentage shown in the axis labels are the percentage of the variance by each principal component; The first number represents a different cultivar, the second number a biological replicate, and the letter “a” or “b” different technical replicates. (**b**) Composition of the first two principal components.

We further analyzed our metabolomic data to determine whether the analyzed cultivars could be grouped according to their metabolomic profiles. For this, we performed a principal component analysis (Figure 5).

The PCA created two principal components, two axes that define a new space, calculated from a linear correlation of the original variables, the metabolite concentrations. The contribution of each metabolite to the new axes, the two new components, is shown in Figure 5b, and the location of the samples in these new axes is shown in Figure 5a. In this projection the cultivars were segregated into three main groups. One group was formed by cultivar 3, and the other group by cultivars 5, 6 and 11, while the remaining cultivars constituted a different group (Figure 5a). We observed that the metabolites negatively correlating with taste (lysine, alanine and leucine) were grouped together. In fact, phenylalanine, which also presented a high degree of correlation (>0.1), appeared in the same group. Importantly, GABA, the metabolite correlating with good taste, appeared isolated and in the opposite sector of the PCA (Figure 5b).

## 4. Discussion

We have previously studied the primary metabolome of broccoli and its relation to abiotic stress. We have found that under salt stress the most limiting factors are the citric acid cycle, as cultivars with higher a content of these components were more tolerant to salt stress [14]. We performed a similar study under drought stress and found that drought-tolerant cultivars had lower amounts of urea, quinic acid, and the gluconic acid lactone. Interestingly drought-stressed broccoli accumulated more essential amino acids. [15]. Having observed that this kind of approach can be used to find the biochemical basis of macroscopic processes, we wanted to use an adaptation of this methodology to investigate the metabolites involved in broccoli taste or flavor.

The pungent taste associated with broccoli has been related to the presence of glucosinolates, a complex family of molecules derived from some amino acids. A recent study showed that glucosinolates are important in the bitter taste perception of Brussels sprouts, given that this concentration is higher than in broccoli. [16]. The influence of glucosinolates in final taste has been observed in other reports, although the same reports found a low variability among cultivars [17].

We wanted to determine which primary metabolites could be related to broccoli taste. For this we compared the metabolomes of 20 different cultivars, grown under field conditions with the taste qualifications assigned by a consumer panel. We used field conditions in order to make the experiment as similar as possible to the conditions in which the standard consumer is going to find broccoli in the supermarket. Even though we used buds of broccoli cultivated in the field and compared 20 different cultivars, most of the metabolites were stable and the variability was very low. Among the metabolites with higher variability, we found sugars, such as glucose of fructose, and the imino acid, proline. These three molecules could present considerable differences among cultivars due to variations in their genetic backgrounds. However, since they are also related to drought stress response [18], we cannot discard that these changes could be due to environmental factors, although we have previously shown that none of these metabolites are limiting for the drought stress response [15].

Surprisingly, we did not find any correlation among those cultivars scoring high in the taste panel and their sugar content. We identified five molecules that presented a high correlation (>0.15) with taste, either negative or positive. Interestingly, three amino acids (lysine, alanine and leucine) negatively correlated with good taste. Alanine is considered to have a sweet taste, similar to saccharine [19], although is able to interact with glutamate receptors [20,21], and so it could have influenced the taste perception. Leucine is known to have a bitter, strong and unpleasant odor [22]. The case for lysine is similar, as its presence has also been related to bad taste [23]. Alanine and leucine are present in high quantities compared to other metabolites, thus reinforcing the idea that their presence is responsible for the low taste scores. Lysine concentrations, on the other hand, are lower than the other two amino acids, and thus its effect on taste perception may not be as important (Figure 1). 

The other metabolite that negatively correlated with broccoli taste was myo-inositol. This molecule has a sweet taste [24], and has been related to good taste in some snacks [25] and sweetness in wine [26]. Myo-inositol is also found in higher concentrations in naturally-ripened kiwi, as compared to exogenous ethylene-induced ripened kiwi [27]. Surprisingly, in our study myo-inositol correlated with bad taste. Other sugars, such as fructose and glucose, were present in relatively high amounts (Figure 1) and were very variable among the cultivars we analyzed (Figure 2). However, we did not find any correlation with taste (Figure 3). This suggests that a positive evaluation of broccoli taste is not related to sweetness and that there are likely to be other molecules competing with or inhibiting the sweet taste or having an antagonistic effect. This is in agreement with a recent report [16] in which it was shown that sweetness plays a significant role in the perception of some Brassica vegetables but not in broccoli.

The only metabolite which presented a positive correlation with taste was GABA, an amino acid not present in proteins. This metabolite has been associated with the development of the sweet-acidic taste in pineapple [28]. It was also shown to correlate with better taste after glycine-betaine treatment in peach [29]. Interestingly GABA has been described as a health-promoting functional compound [30]. Currently, the only gene-edited crop currently commercialized is a tomato cultivar with a higher content of GABA [31]. This crop is available in the Japanese market [32], but there is no published information on its taste compared to non-edited tomato with less GABA content.

In conclusion, by comparing the metabolomic profile of twenty different broccoli cultivars, we have found that amino acids have a pivotal role in determining taste perception. Leucine, alanine and lysine correlate with worse taste, while GABA correlates with better taste. We failed to find a correlation between abundant sugars and positive taste evaluation. In fact, we observed a negative correlation for myo-inositol, suggesting that the sweet taste is not dominant for broccoli. These results can determine future prospects for the classical breeding of new cultivars or biotechnological improvement aiming at less content in leucine, lysine, myo-inositol and alanine and higher content in GABA. This later trait could also further enhance the health-promoting properties of broccoli.

## Data Availability

All data is available in the figures and in the Supplementary Materials.

## Supplementary Materials

The following supporting information can be downloaded at: https://www.mdpi.com/article/10.3390/foods12020339/s1, File S1: complete raw data and histograms for each metabolite in each cultivar. File S2: regression analysis for all the studied metabolites.
